# Myofibroblasts and colonic anastomosis healing in Wistar rats

**DOI:** 10.1186/1471-2482-11-6

**Published:** 2011-03-02

**Authors:** Christophoros Kosmidis, Christoforos Efthimiadis, Georgios Anthimidis, George Basdanis, Stylianos Apostolidis, Prodromos Hytiroglou, Kalliopi Vasiliadou, John Prousalidis, Epameinondas Fahantidis

**Affiliations:** 1 A' Propedeutic Surgical Clinic, Aristotle University Medical School, AHEPA Hospital, Thessaloniki, Greece; 2 Department of Pathology, Aristotle University Medical School, Thessaloniki, Greece

## Abstract

**Background:**

The myofibroblasts play a central role in wound healing throughout the body. The process of wound healing in the colon was evaluated with emphasis on the role of myofibroblasts.

**Methods:**

One hundred male Wistar rats weighing 274 ± 9.1 g (mean age: 3.5 months) were used. A left colonic segment was transected and the colon was re-anastomosed. Animals were randomly divided into two groups. The first group experimental animals (n = 50) were sacrificed on postoperative day 3, while the second group rats (n = 50) were sacrificed on postoperative day 7. Healing of colonic anastomosis was studied in terms of anastomotic bursting pressure, as well as myofibroblastic reaction and expression of α-smooth muscle actin (α-SMA), adhesion formation, inflammatory reaction and neovascularization.

**Results:**

The mean anastomotic bursting pressure increased from 20.6 ± 3.5 mmHg on the 3^rd ^postoperative day to 148.8 ± 9.6 Hg on the 7^th ^postoperative day. Adhesion formation was increased on the 7^th ^day, as compared to the 3^rd ^day. In addition, the myofibroblastic reaction was more profound on the 7^th ^postoperative day in comparison with the 3^rd ^postoperative day. The staining intensity for α-SMA was progressive from the 3rd to the 7th postoperative day. On the 7^th ^day the α-SMA staining in the myofibroblats reached the level of muscular layer cells.

**Conclusions:**

Our study emphasizes the pivotal role of myofibroblasts in the process of colonic anastomosis healing. The findings provide an explanation for the reduction in the incidence of wound dehiscence after the 7th postoperative day.

## Background

Healing of full-thickness injury to the gastrointestinal tract remains an unresolved topic. It begins with a surgical reapposition of the bowel ends, which is most often the initial step in the repair process. Failure of healing results in dehiscence, leaks, and fistulas, which carry significant morbidity and mortality. The myofibroblasts play a central role in the process of wound healing [1-5]. They contain smooth muscle myosin isoforms in addition to α-smooth muscle actin (α-SMA), the requisite machinery for contraction and/or motility, respond to proinflammatory cytokines with elaboration of matrix proteins and additional growth factors and then disappear by apoptosis following repair or scar formation [6-11].

The purpose of this study is to assess the events of colonic anastomosis healing with an emphasis in the role of myofibroblasts.

## Methods

### Experimental Design

The study protocol was approved by the Research Ethics Committee of the Aristotle University of Thessaloniki and conducted in accordance with the Declaration of Helsinki. One hundred male Wistar rats weighing 274 ± 9,1 g (mean age: 3.5 months) were used, which is the least acceptable number for statistical comparison of the groups. The rats were housed two per cage in a standard animal room in the animal laboratory of AHEPA University Hospital, and were allowed free access to food and water before experimentation, so that the integrity of the bowel mucosa was maintained. Rats were anesthetized by intramuscular administration of midazolam (2 mg/kg) (Dormicum Roche K Pharma) and fentanyl (300 μg/kg) (FENTANYL/JANSSEN). Laparotomy was performed through a midline 4 cm incision. A left colonic segment, 1 cm in length, 4 cm proximal to the peritoneal reflection was transected. The colon was re-anastomosed end-to-end using 7-0 propylene sutures (Prolene ETHICON) in single-layer interrupted fashion. Propylene was used as a material because it is a monofilament, nonabsorbable suture. Its advantages include high tensile strength, minimal tissue reactivity, and slipperiness (allowing easy removal from tissues).

To facilitate the suturing, an intraluminal metal tube was inserted transanally at the level of the anastomosis offering absolute apposition of the cut ends. About 10-12 sutures were placed symmetrically for each anastomosis to secure an inverted anastomosis without mucosal protrusion, which is a major cause of perianastomotic adhesions. By this process the operative time was reduced, given that the placement of sutures was expedited and accelerated, while the risk of catching the mucosa of the opposite side was nullified. What is more, the technical uniformity and perfection was ensured, so that the factor of operative technique affected the healing of anastomoses equally.

The tube was removed transanally immediately after the suturing was completed. The abdominal muscle wall was then closed with 4-0 silk sutures, followed by skin closure with 4-0 silk sutures (Mersilk ETHICON). Animals were randomly divided into two groups. The first group experimental animals (n = 50) were sacrificed on postoperative day 3, while the second group rats (n = 50) were sacrificed on postoperative day 7 with an overdose of ether. The previous abdominal incision was reopened, and the anastomotic site identified and inspected for possible adhesions and leakage. A 5 cm segment of the colon with the anastomosis in the middle was resected. Care was taken not to detach adhesions from the anastomosis, but to dissect the surrounding tissues. The resected specimen was gently irrigated with saline to remove feces and was mounted on a table.

Healing of colonic anastomosis was studied in terms of anastomotic bursting pressure, as well as myofibroblastic reaction and expression of α-SMA, adhesion formation, inflammatory reaction and neovascularization. The anastomotic bursting pressure was studied by applying an internal hydraulic pressure (ANNE, Anesthesia Infuser, ABBOTT, USA). As a result, a stress state equivalent to the so-called biaxial tension appeared on the surface of the concave cylindrical specimen (reduced to mmHg, Monitor Minimon 7132 Kontron Instruments, Ltd, England). The level of adhesion formation was determined according to the Van der Ham scale score [12].

### Histopathological and immunohistochemical assessment

A 2 cm-long segment of colon, including the anastomotic site, was resected from each animal, was fixed in 10% buffered formalin for 48 hours, and was submitted for histological examination. Four-cm thick paraffin sections were prepared from each paraffin block using a standard microtome. Sections from each block were stained with hematoxylin-eosin (H&E) and with the streptavidin-biotin immunohistochemical procedure for alpha-smooth muscle actin (α-SMA). The antiserum was obtained from Dakopatts, Glostrup, Denmark. In the bowel, α-SMA is expressed in smooth muscle fibers of the muscularis mucosae and the muscularis propria, as well as smooth muscle fibers and pericytes of vascular walls. In areas of wound healing, myofibroblasts also show α-SMA immunoreactivity.

The histological and immunohistochemical assessment were performed by a "blinded" observer, a pathologist who was unaware of the study groups. The following histological findings were assessed semiquantitatively as mild = +, moderate = ++ or severe = +++: density of inflammatory reaction, intensity of myofibroblastic reaction and density of neovascularization. The density of the inflammatory infiltrates, as well as the density of the new vessels were assessed in H&E-stained sections. The intensity of the myofibroblastic reaction was evaluated in immunohistochemical stains for α-SMA as follows: +: loosely arranged myofibroblasts; ++: moderately dense myofibroblasts; +++: densely arranged myofibroblasts. All examinations were performed at an Olympus BH2 microscope, at magnifications of ×100 and ×400.

### Statistical Analyses

All data were recorded using Statistical Package for the Social Sciences (SPSS) 16 for Windows. An independent-samples t-test was conducted to compare the bursting pressure for the two groups of experimental animals. The Chi-square test for independence was used to find out whether there was a relationship between group of rats and adhesion formation, inflammatory reaction, myofibroblastic reaction and neovascularization. *P *values below 0.05 were considered significant.

## Results

The mean anastomotic bursting pressure increased from 20.6 ± 3,5 mmHg on the 3^rd ^postoperative day to 148.8 ± 9.6 mmHg on the 7^th ^postoperative day. There was statistically significant difference in bursting pressure between the two groups of rats (p < 0.001). The magnitude of the differences in the means was very large (eta squared = 0.987). Interestingly, wound rupture was located at the anastomotic site, when rats were sacrificed on the 3^rd ^postoperative day. On the other hand, wound breakdown took place at the wound margin, when animals were sacrificed on the 7^th ^postoperative day. Adhesion formation according to Van der Ham scale score is illustrated in Table 1. There was a statistically significant relationship between group of rats and adhesion formation (p = 0.007).

**Table 1:** van der Ham scale for adhesion formation score

			Adhesion formation			Total
				
			0	I	II	
**group**	**3rd day**	Count	5	30	15	50
		
		% of Total	5.0%	30.0%	15.0%	50.0%
	
	**7th day**	Count	5	15	30	50
		
		% of Total	5.0%	15.0%	30.0%	50.0%

Total	Count	10	45	45	100	
	
	% of Total	10.0%	45.0%	45.0%	100.0%	

Van der Ham adhesion scoring system:

- No adhesion

+ Adhesion towards the anastomosis line from the omentum

++ Adhesion of bowel and omentum towards the anastomosis line

+++ Extensive adhesions towards the anastomosis line and contemporary presence of abscess

The granulation tissue at the anastomotic area on the 3^rd ^postoperative day was rich in polymorphonuclear leukocytes, lymphocytes and histiocytes. The stroma was edematous and contained many capillary vessels. The intensity of the inflammatory reaction detected was less on the 7^th ^postoperative day, as compared to the 3^rd ^postoperative day (Table 2). On the 7^th ^postoperative day, lymphocytes and histiocytes were the predominant cells in the granulation tissue, which also contained prominent myofibroblasts. The Chi-Square test showed that there was no statistically significant relationship between group of rats and inflammatory reaction (p = 0.356). The neovascularization was less intense on the 7^th ^postoperative day as compared with the 3^rd ^postoperative day (Table 3). There was no statistically significant relationship between group of rats and neovascularization (p = 0.108).

**Table 2:** Inflammatory reaction at the anastomosis site.

			Inflammatory reaction		Total
	
			+	++	
**Group**	**3rd day**	Count	10	40	50
		
		% of Total	10.0%	40.0%	50.0%
	
	**7th day**	Count	15	35	50
		
		% of Total	15.0%	35.0%	50.0%

Total		Count	25	75	100
		
		% of Total	25.0%	75.0%	100.0%

**Table 3:** Neovascularization at the anastomosis site.

			neovascularization			Total
	
			+	++	+++	
**group**	**3rd day**	Count	5	40	5	50
		
		% of Total	5.0%	40.0%	5.0%	50.0%
	
	**7th day**	Count	13	32	5	50
		
		% of Total	13.0%	32.0%	5.0%	50.0%

Total		Count	18	72	10	100
		
		% of Total	18.0%	72.0%	10.0%	100.0%

**Figure 1:**
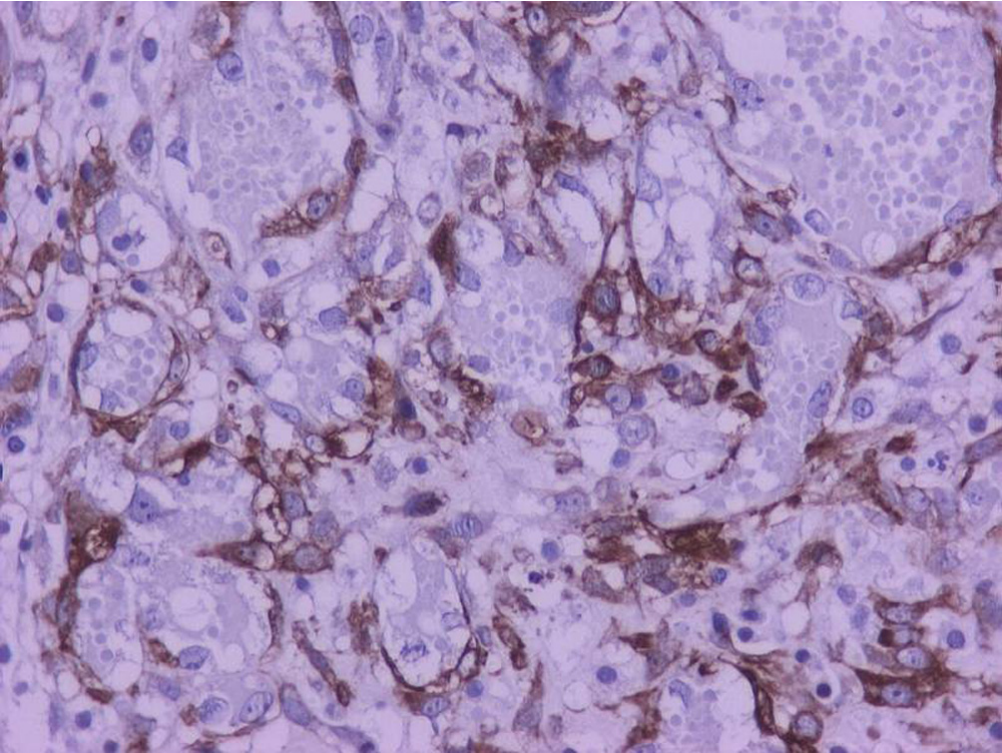
**Expression of α-SMA in anastomotic sites: The antigen is expressed in myofibroblasts, pericytes and smooth muscle cells (3^rd ^postoperative day)**. The staining intensity of myofibroblasts is weaker than that of muscularis propria fibers (immunohistochemical stains for α-SMA, ×400).

**Figure 2:**
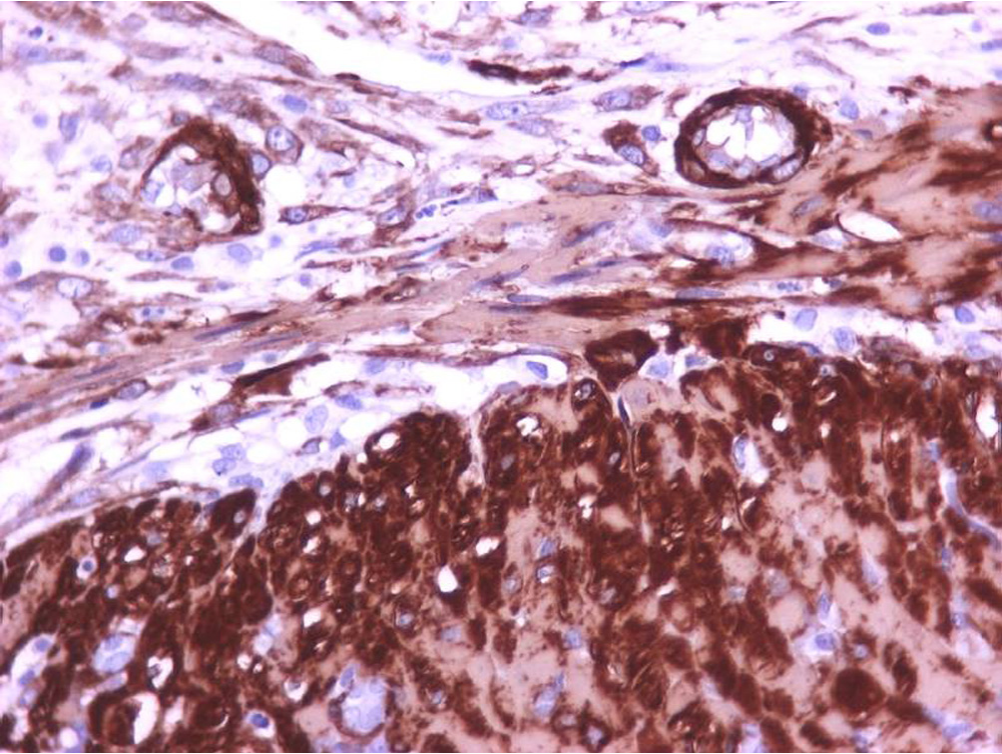
**Bigger magnification of the anastomotic site (3^rd ^postoperative day) (immunohistochemical stains for α-SMA, ×400)**.

**Figure 3:**
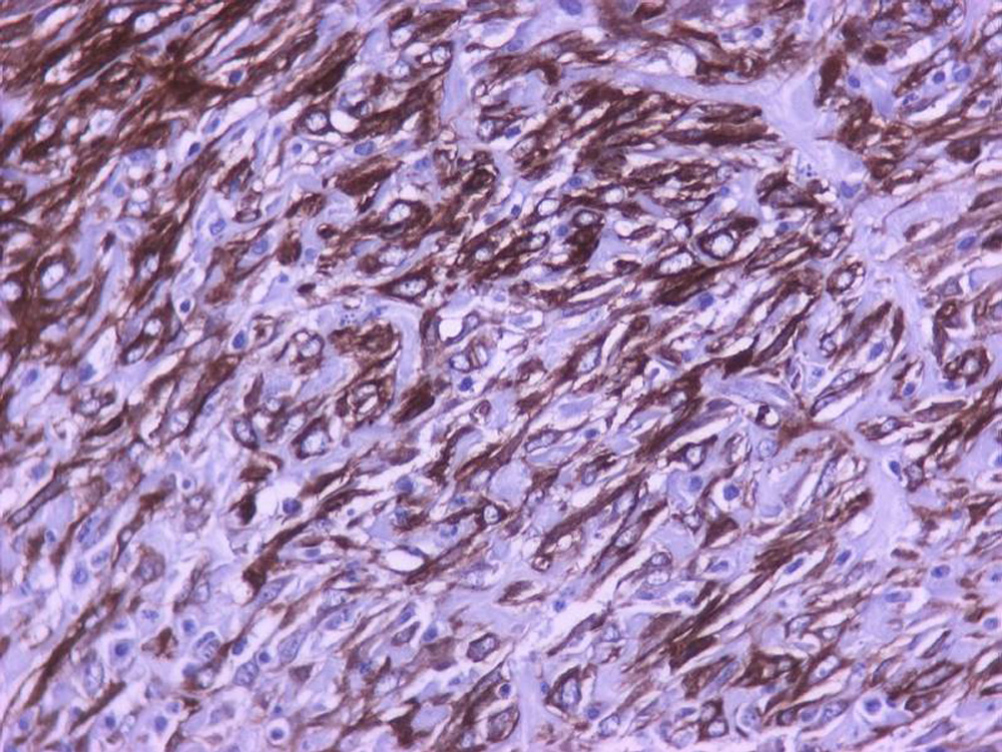
**On the 7^th ^postoperative day, the α-SMA content of the myofibroblasts is similar to that of muscular layer cells (immunohistochemical stains for α-SMA, ×400)**.

**Figure 4:**
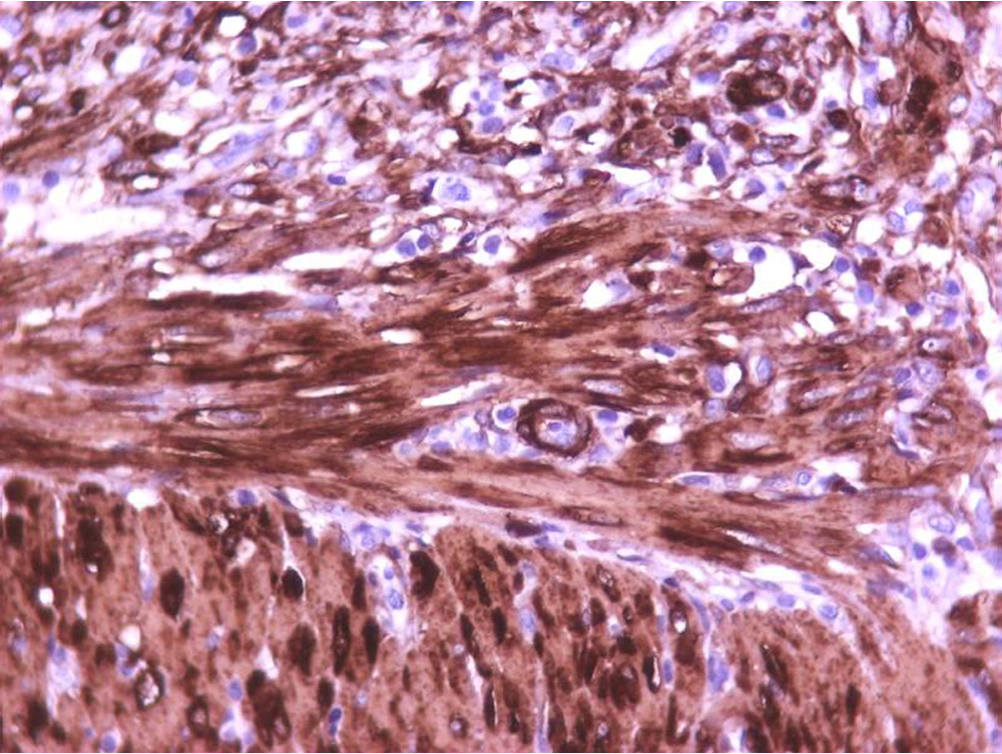
**Bigger magnification of the anastomotic site (7^th ^postoperative day) (immunohistochemical stains for α-SMA, ×400)**.

The myofibroblastic reaction was more profound on the 7^th ^postoperative day in comparison with the 3^rd ^postoperative day (Table 4). There was a statistically significant relationship between group of rats and myofibroblastic reaction (p < 0,01). In addition to myofibroblasts, α-SMA was expressed in pericytic and smooth muscle cells of old and newly formed vessels, as well as muscle fibers of the muscularis mucosae and the muscularis propria. The staining intensity of the myofibroblasts increased from the 3rd to the 7th postoperative day. On the 7^th ^day, the α-SMA content of the myofibroblasts reached the level of the muscular layer cells (Figures 1, 2, 3, 4). Anastomotic leakage or other complications that might correlate to the histological and immunohistological findings were not observed.

**Table 4:** Myofibroblastic reaction at the anastomosis site.

			myofibroblasts			Total
	
			+	++	+++	
**group**	**3rd day**	Count	35	15	0	50
		
		% of Total	35.0%	15.0%	.0%	50.0%
	
	**7th day**	Count	15	10	25	50
		
		% of Total	15.0%	10.0%	25.0%	50.0%

Total		Count	50	25	25	100
		
		% of Total	50.0%	25.0%	25.0%	100.0%

## Discussion

Dehiscence of colonic anastomosis is a common, serious and potentially life-threatening complication after colorectal operation. The early integrity of the anastomosis is dependent on formation of a fibrin seal on the serosal side, which achieves watertightness, and on the suture-holding capacity of the intestinal wall, particularly the submucosal layer [13]. A key event in the process of wound repair is contraction of the gastrointestinal lamina propria [4,6,14-17]. Ultimate anastomotic strength in the gastrointestinal tract is not always related to the absolute amount of collagen, and the structure and arrangement of the collagen matrix may be more important [18]. Myofibroblasts appear to be key cells in the process of wound healing. They are the major responsible cells for contraction and are also involved in the formation and repair of the extracellular matrix and proliferation and differentiation of epithelial, vascular and neurogenic elements [13,19-22].

The integrity of the anastomosis represents equilibrium between collagen lysis, which occurs early in the healing process, and collagen synthesis, which takes a few days to initiate. During the first 3 to 5 days collagen breakdown far exceeds collagen synthesis. There is a significant decrease in marginal strength during the first week due to an early and marked collagenolysis [13,18]. Local infection, which often occurs near colonic anastomoses, promotes lysis and delays synthesis, thus increasing the likelihood of perforation. The danger of leakage is most from the fourth to seventh days, when tensile strength normally would rise rapidly but is prevented from doing so by increased lysis or compromised collagen deposition [23].

Leakage is about as likely to occur a few millimeters from the anastomosis as it is in the anastomosis itself. By 1 week intestinal anastomosIs resists bursting more strongly than the more normal surrounding intestine. This has been attributed to the fact that the surrounding intestine also participates in the reaction to injury and loses a large part of its collagen by lysis [23]. However, one could argue that although collagen lysis occurs both at the anastomosis and the surrounding intestine, the wound margin is intact, while the anastomotic site is cut and forming anew. Our study demonstrated that wound rupture was located at the anastomotic site on the 3^rd ^postoperative day while wound breakdown took place at the wound margin on the 7^th ^postoperative day.

The immunohistochemical findings our study expand those of Darby et al. [6] on experimental wound healing. Darby et al examined skin wounds in rats by immunofluoresence and found that α-SMA started to become detectable in myofibroblasts on day 6, and then was increasingly expressed for the following 15 days of wound healing [6]. On the other hand, the contemporary immunohistochemical procedure that was utilized in our study showed that α-SMA immunoreactive myofibroblasts were evident at the anastomosis already on the 3rd postoperative day. By the 7th day, the number of cells with positive α-SMA expression increased. Actually, the immunohistochemical study of specimens showed that the content of myofibroblasts in α-SMA was remarkably increased by the 7^th ^day, in fact, reaching the level of the muscular layer cells. This finding provides an explanation for the reduction in the incidence of wound dehiscence after the 7th postoperative day. Moreover, it could explain why by 1 week the proportion of wound dehiscence that occurs at the anastomotic site is not expectedly more than that at the wound margin.

Our results are supported by the fact that wound contraction begins immediately after injury. This had been considered to be a puzzling point, since it did not correspond to the day (6^th^) myofibroblasts were identified in the wound. It had been attributed to the in vitro findings of Ehrlich that fibroblasts placed in a collagen lattice actively move in the lattice and contract it without expressing stress fibers, postulating that the movement of cells with concomitant reorganization of the cytoskeleton is responsible for contraction [6,13,24].

## Conclusions

The potential to minimize failure of wound healing depends on the surgeon's knowledge of the events responsible for this phenomenon. The results of our study emphasize the pivotal role of myofibroblasts in the process of colonic anastomosis healing and provide an explanation for the reduction in the incidence of wound dehiscence after the 7th postoperative day. Further understanding of the molecular mechanisms of normal and pathologic wound healing may provide valuable insights into future therapies that can control dehiscence, leaks, and fistulas.

## Competing interests

The authors declare that they have no competing interests.

## Authors' contributions

CK carried out the experimental studies and drafted the manuscript, made substantial contributions to conception and design, acquisition of data, analysis and interpretation of data.

CE participated in the experimental studies.

GA performed the statistical analysis  involved in revising critically the manuscript for important intellectual content.

GB participated in the design of the study and made substantial contributions to interpretation of data.

SA made substantial contributions to  acquisition of data and the study of the anastomotic bursting pressure.

PH performed the histological and immunohistochemical assessment  involved in revising critically the manuscript for important intellectual content.

KV made substantial contributions to collecting references, designing and revising data of the paper and resubmitting it.

JP conceived of the study, and participated in its design and coordination.

EF conceived of the study, and participated in its design and coordination.

All authors read and approved the final manuscript.

## Pre-publication history

The pre-publication history for this paper can be accessed here:

http://www.biomedcentral.com/1471-2482/11/6/prepub
